# Effects of Eleclazine (GS6615) on the proarrhythmic electrophysiological changes induced by myocardial stretch

**DOI:** 10.3389/fphys.2025.1525836

**Published:** 2025-01-31

**Authors:** Francisco J. Chorro, Luis Such-Miquel, Samuel Cuñat, Oscar Arias-Mutis, Patricia Genovés, Manuel Zarzoso, Antonio Alberola, Luis Such-Belenguer, Irene Del Canto

**Affiliations:** ^1^ Department of Medicine, University of Valencia, Valencia, Spain; ^2^ Biomedical Research Center Network - Cardiovascular Diseases (CIBERCV), Carlos III Health Institute, Madrid, Spain; ^3^ Research Institute, Valencia Clinic Hospital (INCLIVA), Valencia, Spain; ^4^ Department of Physiotherapy, University of Valencia, Valencia, Spain; ^5^ Department of Physiology, University of Valencia, Valencia, Spain; ^6^ Department of Electronic Engineering, University of Valencia, Burjassot, Spain

**Keywords:** arrhythmias, myocardial stretch, Eleclazine (GS6615), ventricular fibrillation, cardiac mapping

## Abstract

**Purpose:**

Myocardial stretch is a proarrhythmic factor. Eleclazine (GS6615) is a late sodium current (INaL) inhibitor that has shown protective effects against arrhythmias in various experimental models. Data on its effects during myocardial stretch are lacking. The aim of this study was to investigate the electrophysiological modifications induced by eleclazine basally and during acute ventricular stretch.

**Methods:**

Left ventricular stretch was induced at baseline and during perfusion with eleclazine in 26 Langendorff rabbit heart preparations. Programmed stimulation and high-resolution mapping techniques were applied using multiple epicardial electrodes.

**Results:**

At baseline, both the ventricular refractory period measured at a fixed cycle length (250 m) and its surrogate obtained during ventricular fibrillation (VF) decreased significantly during stretch (baseline 128 ± 15 vs. stretch 110 ± 14 m; n = 15; p < 0.001, and baseline 52 ± 13 vs. stretch 44 ± 9 m; n = 11; p < 0.05), while the VF dominant frequency (DF) increased significantly (DF baseline 13 ± 3 vs. stretch 17 ± 5Hz; n = 11; p < 0.01). Eleclazine 1.4 μM prolonged refractoriness, diminished both DF and conduction velocity during the arrhythmia, and avoided the stretch induced variations in refractoriness (baseline 148 ± 19 vs. stretch 150 ± 23 m; n = 15; ns, and baseline 73 ± 15 vs. stretch 77 ± 15 m; n = 11; ns) and in DF (baseline 12 ± 5 vs. stretch 12 ± 3 Hz; ns). The VF complexity index was inversely related to refractoriness (r = −0.64; p < 0.001). Under eleclazine perfusion, the VF activation patterns were less complex, and the arrhythmia stopped in 6 out of 11 experiments (55%; p < 0.05 vs. baseline).

**Conclusion:**

Eleclazine (GS6615) reduced the proarrhythmic electrophysiological changes induced by myocardial stretch and slowed and simplified activation patterns during VF in the experimental model used.

## 1 Introduction

Cardiac arrhythmias represent a major public health concern worldwide. Related clinical manifestations such as sudden cardiac death constitute an important challenge, with the need to develop lines of research aimed at better understanding the underlying causes, mechanisms and the most effective preventive and therapeutic approaches (Marijon et al., 2023; Lynge et al., 2021; Lynge et al., 2023; Jain et al., 2024; Chatterjee, 2023). Its importance is reflected in initiatives such as that of the Lancet commission, seeking to reduce the global burden of sudden cardiac death, which highlights the need for multidisciplinary approaches (Marijon et al., 2023). The mechanisms involved in the appearance of cardiac arrhythmias are diverse and are related, among other factors, to modifications of both the substrate that supports them and the presence of triggering factors (Chatterjee, 2023; Lerman et al., 2024; Packer, 2020). Mechanical stretch acts on both components, and its effects are part of so-called mechanoelectric feedback (Quinn and Kohl, 2021; Sutherland, 2017; Johnson and Antoons, 2018; Gottlieb et al., 2023). Mechanical forces modify cellular electrophysiology through the activation and/or modulation of transmembrane ionic channels and alterations in intracellular calcium handling (Quinn and Kohl, 2021; Ravelli and Allessie, 1997; Chorro et al., 2005; Chorro et al., 2009; Reiter et al., 1988; Prosser et al., 2013). These effects favor the production of afterpotentials and the induction of reentrant activations responsible for the appearance of arrhythmias, as extensively reviewed by several authors (Quinn and Kohl, 2021; Sutherland, 2017; Johnson and Antoons, 2018).

Previous studies have shown the attenuation of the electrophysiological effects induced by mechanical stretch by Na^+^/H^+^ exchanger inhibitors such as EIPA (Chorro et al., 2015a), Na^+^/Ca^2+^ inhibitors such as KB-R7943 (Chorro et al., 2009) or betablockers such as propranolol (Chorro et al., 2009). Eleclazine (GS6615) is an INaL inhibitor (Rajamani et al., 2016; Zablocki et al., 2016; Liu et al., 2023) that has shown protective effects against arrhythmias in various experimental models (Rajamani et al., 2016; Zablocki et al., 2016; Liu et al., 2023; Fuller et al., 2016; Bacic et al., 2017; Caves et al., 2020; Shen et al., 2022). However, a reduction in arrhythmic complications has not been demonstrated in clinical studies (Shen et al., 2022; Olivotto et al., 2016). Thus, in patients with ventricular tachycardia/ventricular fibrillation treated with implantable cardioverter-defibrillators, eleclazine failed to show a significant reduction in appropriate interventions, and the clinical trials designed to evaluate the effect of eleclazine on symptoms and exercise capacity in patients with hypertrophic cardiomyopathy and to evaluate the safety and tolerability of the drug in patients with long QT syndrome type 3 were discontinued. Experimental data on its effects in other proarrhythmogenic contexts as myocardial stretch are lacking and its analysis can provide data of interest, since other INaL inhibitors such as Ranolazine and GS967 (Del-Canto et al., 2020; Chorro et al., 2015b; Milberg et al., 2013; Del Canto et al., 2018) attenuate these effects. The mechanisms involve both the inhibitory action on INaL and their actions on the rapid component of the sodium current (Burashnikov and Antzelevitch, 2017) and other ionic currents, such as the rapidly activated delayed rectifier potassium current (IKr) (Antzelevitch et al., 2004). Eleclazine is a more potent and selective INaL inhibitor and the study of its actions in this context can provide interesting data on the regulation of the electrophysiological effects produced by mechanical stretch. Myocardial stretch is characterized by an increase in Na + inflow, the activation of the reverse mode of the Na^+^/Ca^2+^ exchanger and an increase in intracellular Ca^2+^ that, together with changes in the oxidative balance, contribute to the activation of INaL. The activity of the sarcoplasmic reticulum, its Ca^2+^ content and the probability of opening the Ryanodine receptor are also altered, favoring the spontaneous release of Ca^2+^. Thus, in order to analyze whether a protective role is present in these settings the present study examines the effects of eleclazine in an experimental model in which the modifications of ventricular refractoriness, conduction velocity, wavelength of the activation process and activation patterns during ventricular fibrillation (VF) are analyzed both basally and during the application of acute mechanical stretch in the left ventricle.

## 2 Materials and methods

The present study followed European Union guidelines (2010/63) and Spanish standards (RD53/2013) on the protection of animals used for scientific purposes. The work was approved by the local Institutional Committee for Animal Care and Use (2015/VSC/PEA/00233 type 2).

### 2.1 Experimental protocol

We used isolated and perfused heart preparations from New Zealand rabbits with a mean weight of 4.5 ± 0.4 kg (Langendorff technique) for the experiments (n = 26). Following the intravenous administration of heparin and thiopental (60 mg per kg), the heart was removed, submerged in cold Tyrode, and subsequently connected to the system via the aorta. The Tyrode was perfused at 37°C ± 0.5°C, the pH was maintained at 7.4, and oxygenation and pH support of the solution were carried out with carbogen (O2: 95% and CO2: 5%). Mean pressure was set at 70 mmHg. A MapTech device (Waalre, Netherlands) and multiple electrode (121 unipolar electrodes, spacing = 1 mm) located on the surface of the anterior wall of the left ventricle were used to record electrophysiological signals. The indifferent electrode was placed over the aorta. Electrical stimuli (duration = 2 m; double intensity of the diastolic threshold) were applied with a Grass S88 stimulator). In order to induce local stretching of the left ventricular free wall, an L-shaped device was introduced into this camera through the left atrium (Chorro et al., 2009; Chorro et al., 2015a). The device consisted of a hollow tube measuring 3.5 mm in diameter, through which a stem 1.5 mm in diameter could be advanced. The distal end of the stem protruded from the L-shaped device and consisted of a circular platform measuring 7.5 mm in diameter, with which controlled stretching of a circumscribed zone of ventricle wall could be induced. A 12% increase in the vertical and transverse axes of the stretched area was obtained by moving (6 mm) the support of the circular platform located in the ventricular cavity. The L-shaped device was introduced before the stabilization period prior to the electrophysiological protocol.

### 2.2 Electrophysiological protocol and parameters analyzed

After 30 min of stabilization, programmed stimulation protocols were applied at baseline, during the perfusion of eleclazine at three concentrations (in a range around the IC50 value for late Na + current blockade [0.35 μM, 0.7 μM, and 1.4 μM]) (Rajamani et al., 2016), and after drug washout. The concentrations were chosen based on the information provided by other studies such as that of Rajamani et al. (2016) who in rabbit ventricular myocytes reported an IC50 value of 0.72 ± 0.06 μM at a holding potential of −120 mV and 0.26 ± 0.01 μM at a holding potential of −80 mV. In the study by Bacic et al. (2017) in which a porcine model was used, they observed that eleclazine administered at a dose of 0.3 mg/kg significantly reduced the incidence of VT in isolated hearts and the plasma levels of the drug were around 0.5 μM. The extrastimulus test was applied to obtain ventricular refractory periods during a fixed cycle length (250 m) in a series of 15 experiments. Ventricular pacing was performed at increasing frequencies to induce ventricular fibrillation in another series of 11 experiments. Coronary perfusion was maintained during the arrhythmia. In each phase, the parameters to be studied were obtained before stretch, 3 min after the start of its application and after stretch suppression.

The parameters analyzed were ventricular refractory periods, conduction velocity, mean and fifth percentile of VV intervals during VF (P5), VF dominant frequency (DF) and spectral concentration (SpC), and characteristics of the activation maps during arrhythmia. The DF is the greatest amplitude in the spectral power distribution of the signal, and SpC is the percentage of the spectrum area in the DF ± 0.5 Hz range. Activation times in each electrode were determined by identifying the moment of maximum negative slope of the electrograms. The minimum threshold for dV/dt to be judged as a local deflection was a percentage (20%) of the maximal negative slope in each channel. The fibrillation interval (VV) histograms and the mean of the consecutive VV intervals were determined during 2-s time windows. Spectral parameters, the VV intervals histograms and the mean value of the consecutive VV intervals were determined for the recordings obtained with all 121 unipolar electrodes of the multiple electrode, during time windows of 2 s. The activation maps during VF were constructed after determining the local activation times and corresponding isochrones during time windows of 1 s. These maps were classified by complexity into types I (a single activation front without lines of conduction block), II (two fronts or one with block lines), and III (three or more fronts with block lines). The conduction velocity (CV) was determined by dividing the distance between two electrodes (spaced 5 mm apart) by the difference between the activation times (average of 5 determinations). During VF, this parameter was determined on the wavefronts where both arrival and exit were recorded in the analyzed area, and to determine the wavelength, the conduction velocity was multiplied by P5 (Chorro et al., 2005; Chorro et al., 2015a).

### 2.3 Statistical analysis

Continuous variables were reported as the mean ± standard deviation (SD), and categorical variables were reported as percentages. The general linear model was used to compare the differences in repeated measures, using Bonferroni’s correction in multiple comparisons. Differences with p < 0.05 values were considered to be statistically significant. The differences between qualitative variables were analyzed using contingency tables and Fisher’s exact test when indicated. Calculations were made using the SPSS version 22.0 statistical package.

## 3 Results

### 3.1 Ventricular refractoriness

**FIGURE 1:**
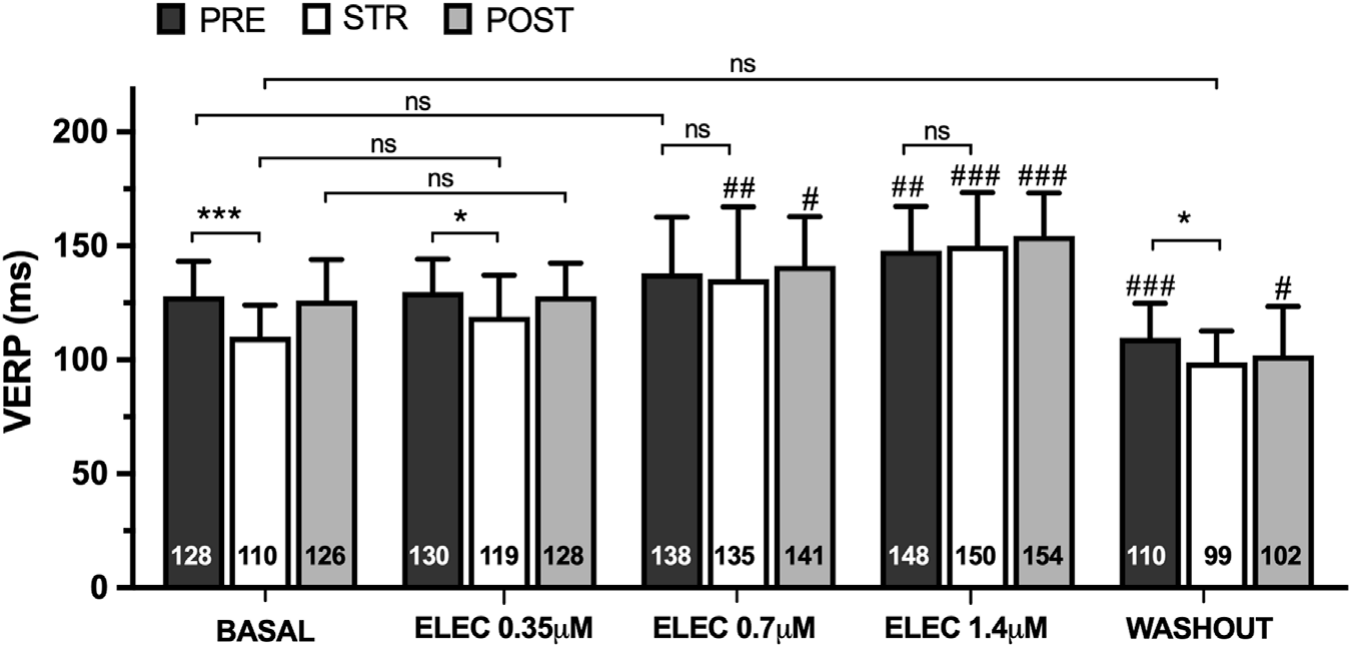
Mean ± standard deviation of ventricular effective refractory period (VERP) obtained at baseline, during the perfusion of eleclazine (ELEC) 0.35 μM, 0.7 μM and 1.4 μM, and after drug washout before (PRE), during (STR) and after (POST) stretch. PRE vs. STR differences: *p < 0.05; ***p < 0.001. Differences vs. baseline: #p < 0.05; ##p < 0.01; ###p < 0.001.

**FIGURE 2:**
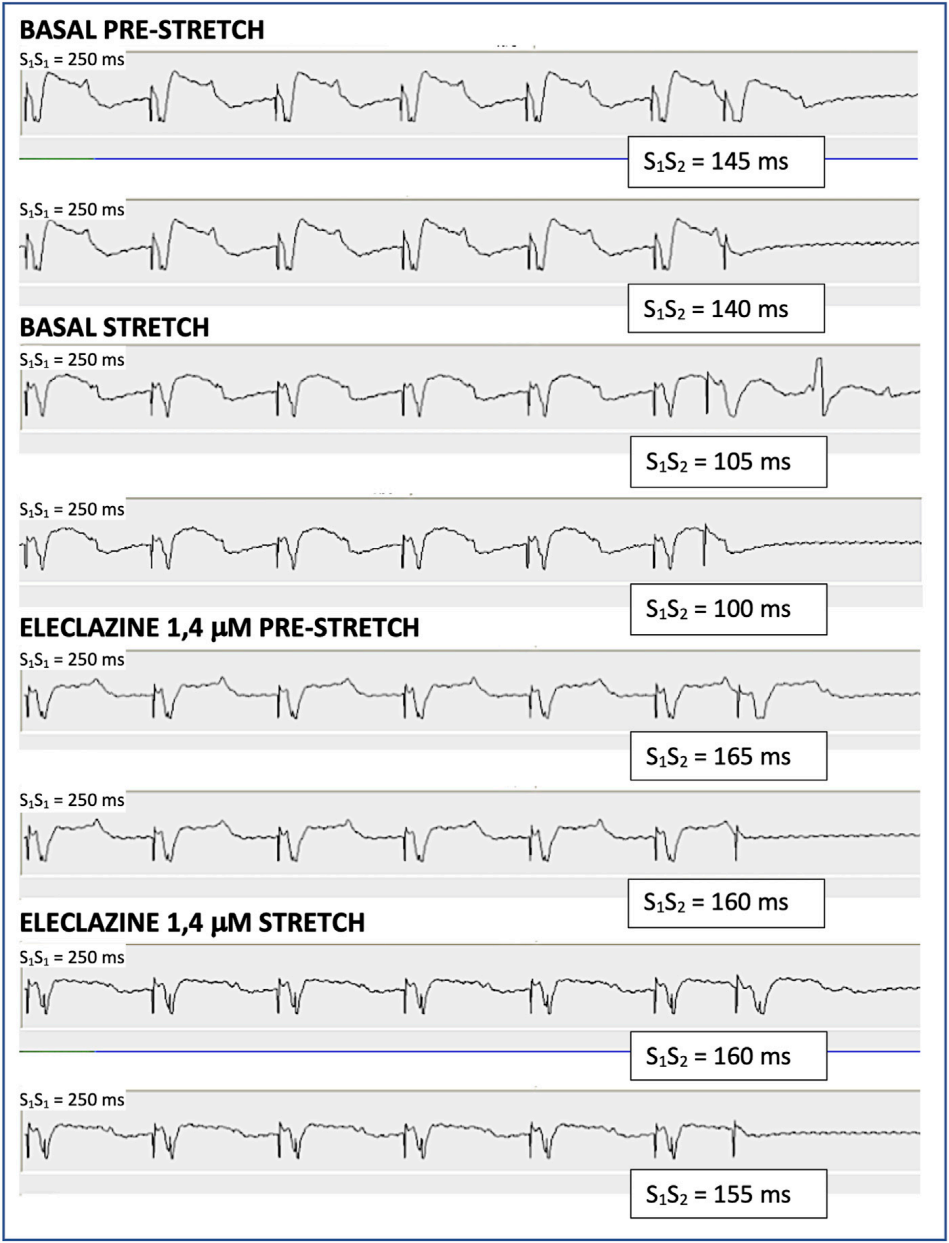
Determination of the ventricular effective refractory period through the extrastimulus test in one of the experiments. S1 are the stimulus of the basic train and S2 is the extrastimulus. The maximum S1S2 interval that does not capture is the refractory period. During baseline myocardial stretch shortens the refractory period by 40 m, while during the perfusion of eleclazine 1.4 μM the refractory period increases and the shortening produced by myocardial stretch is 5 m.

The ventricular effective refractory period (VERP) (n = 15) obtained prior to, during, and following myocardial stretch are displayed in Figure 1. Stretch caused a significant decrease in VERP at baseline (pre 128 ± 15 vs. stretch 110 ± 14 m, p < 0.001) and during eleclazine perfusion at 0.35 μM (pre 130 ± 15 vs. stretch 119 ± 18 m, p < 0.05), while the variation did not reach statistical significance at concentrations of 0.7 μM (pre 138 ± 25 vs. stretch 135 ± 32 m, ns) or 1.4 μM (pre 148 ± 19 vs. stretch 150 ± 23 m, ns) (Figure 2). A significant increase in this parameter was observed compared with baseline during perfusions of 0.7 μM and 1.4 μM. In the final experimental phase (after drug washout), refractoriness was reduced compared with the baseline values, and stretch again caused a significant decrease in VERP (pre 110 ± 15 vs. stretch 99 ± 14 m, p < 0.05).

**FIGURE 3:**
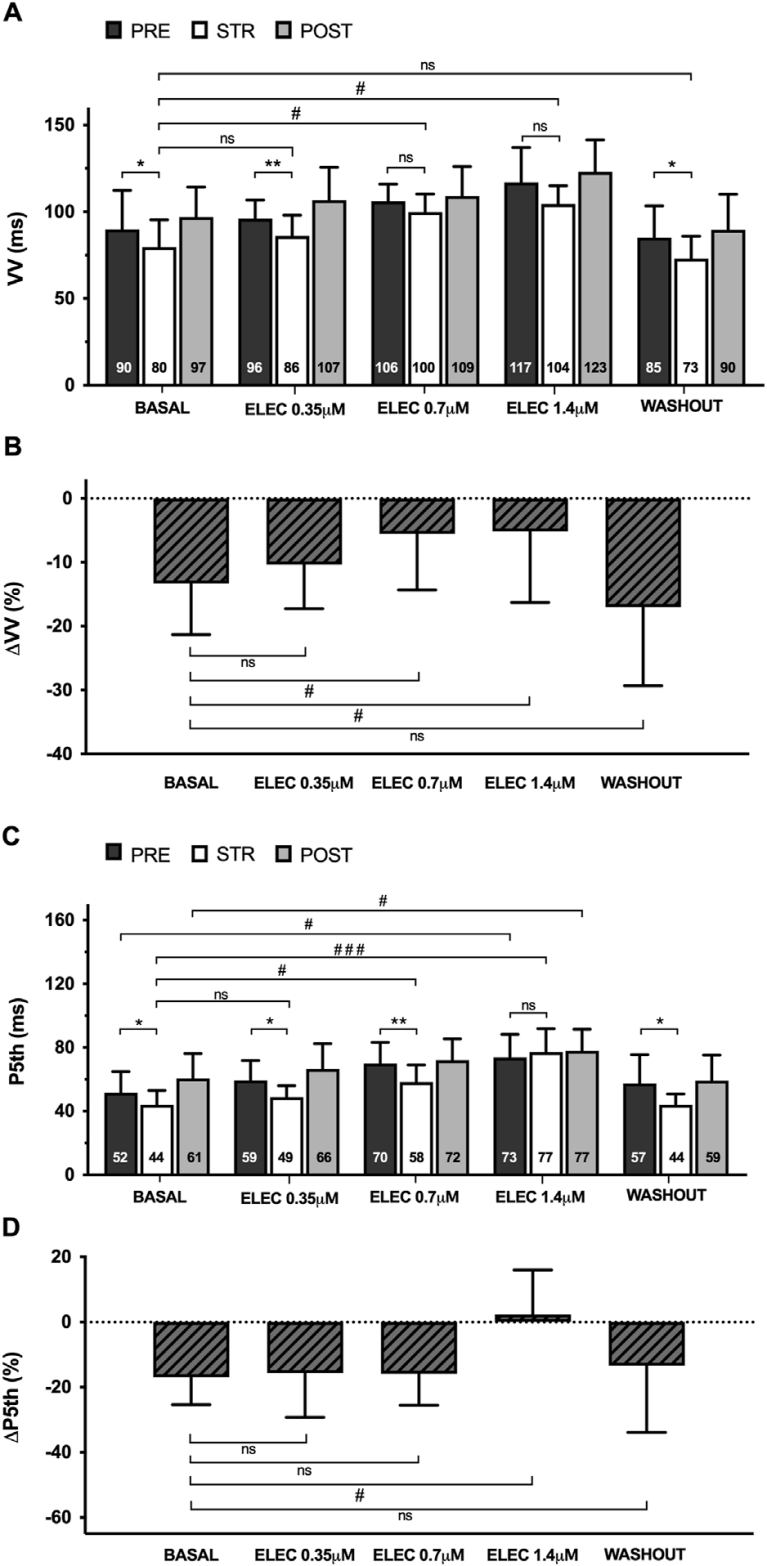
Mean ± standard deviation of the VV **(A)** and fifth percentile intervals (P5th) **(C)** during ventricular fibrillation obtained at baseline, during 0.35 μM, 0.7 μM and 1.4 μM eleclazine perfusion (ELEC), and after drug washout before (PRE), during (STR) and after (POST) stretch. The percentage variations of these parameters produced by stretch (ΔVV and ΔP5th) [**(B, D)** respectively] are also shown. PRE vs. STR differences: *p < 0.05; **p < 0.01. Differences vs. baseline: #p < 0.05; ###p < 0.001.

During pacing-induced ventricular fibrillation (VF) (n = 11), myocardial stretch resulted in a significant decrease in successive activation intervals (VV), both at baseline (pre 90 ± 22 vs. stretch 80 ± 16 m, p < 0.05) and during the perfusion of eleclazine 0.35 μM (pre 96 ± 11 vs. stretch 86 ± 12 m, p < 0.01). Once again, however, this reduction did not reach statistical significance at concentrations of 0.7 μM (pre 106 ± 10 vs. stretch 100 ± 10 m, ns) or 1.4 μM (pre 117 ± 20 vs. stretch 104 ± 10 m, ns) (Figure 3). The same figure shows the variations in fifth percentile of the VV intervals (P5) produced by stretch. This parameter is a surrogate indicator of refractoriness during arrhythmia (Chorro et al., 2005; Chorro et al., 2015a), and differences proved significant at baseline (pre 52 ± 13 vs. stretch 44 ± 9 m, p < 0.05) and under the action of eleclazine 0.35 μM (pre 59 ± 12 vs. stretch 49 ± 7 m, p < 0.05) and 0.7 μM (pre 70 ± 13 vs. stretch 58 ± 11 m, p < 0.01), but failed to reach statistical significance at a concentration of 1.4 μM (pre 73 ± 15 vs. stretch 77 ± 15 m, ns). At this concentration, a significant increase in this parameter with respect to the baseline values was recorded in the three situations studied. After drug washout, P5 decreased, and the reducing effects of stretch on this parameter were once again significant (pre 57 ± 18 vs. stretch 44 ± 7 m, p < 0.05).

### 3.2 VF spectral analysis

**FIGURE 4:**
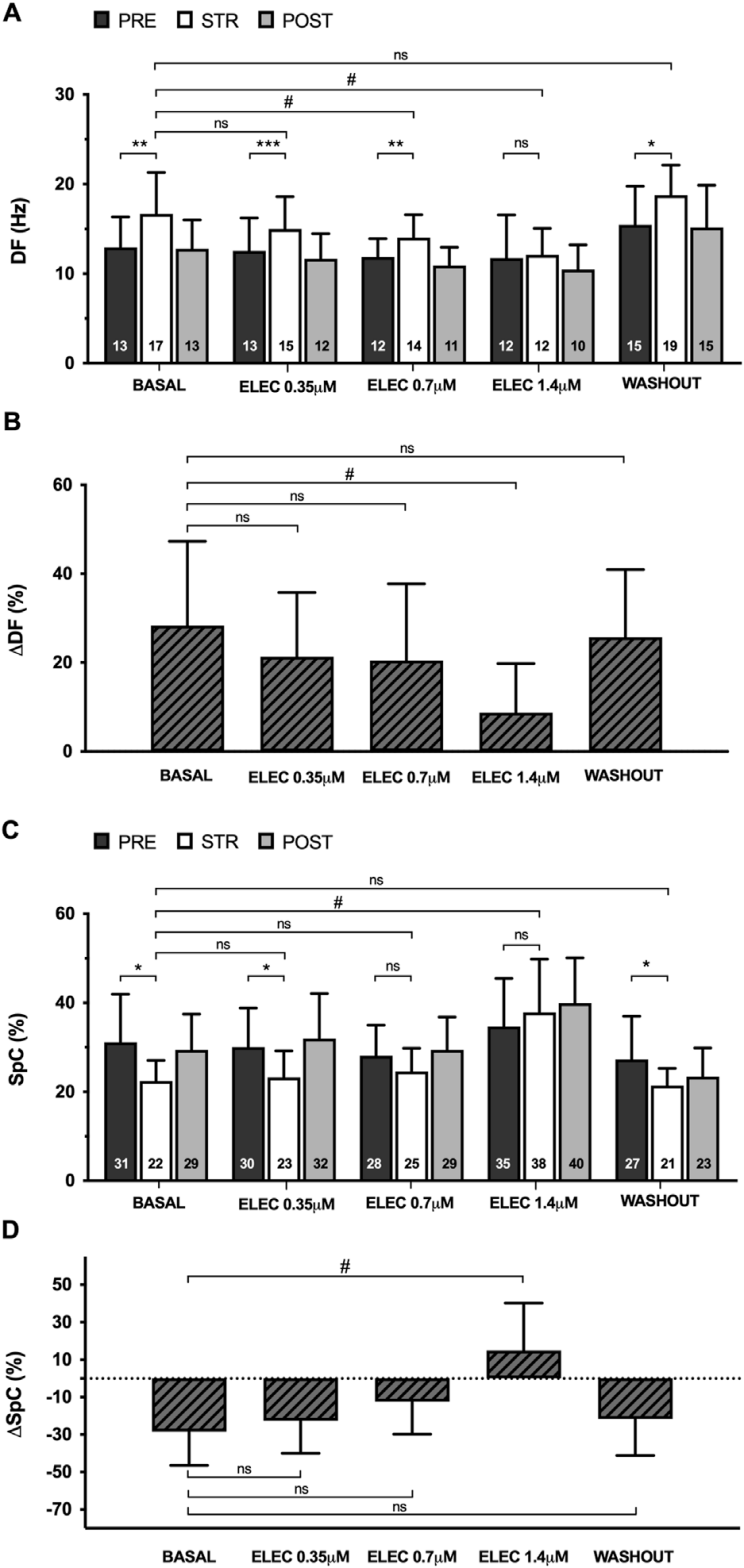
Mean ± standard deviation of dominant frequency (DF) **(A)** and spectral concentration (SpC) **(C)** during ventricular fibrillation obtained at baseline, during 0.35 μM, 0.7 μM and 1.4 μM eleclazine perfusion (ELEC) and after drug washout before (PRE), during (STR) and after (POST) stretch. The percentage variations of these parameters produced by stretch (ΔDF and ΔSpC) [**(B, D)** respectively] are also shown. PRE vs. STR differences: *p < 0.05; **p < 0.01; ***p < 0.001. Differences vs. baseline: #p < 0.05.

Myocardial stretch caused a significant increase in VF dominant frequency (DF) compared to the pre- and post-stretch values, both at baseline (pre 13 ± 3 vs. stretch 17 ± 5 Hz, p < 0.01) and during the perfusion of eleclazine 0.35 μM (pre 13 ± 4 vs. stretch 15 ± 4 m, p < 0.001) and 0.7 μM (pre 12 ± 2 vs. stretch 14 ± 3 m, p < 0.01); the variation was not statistically significant at the 1.4 μM concentration (pre 12 ± 5 vs. stretch 12 ± 3 Hz, ns), however (n = 11) (Figure 4). Under the action of eleclazine (0.7 μM and 1.4 μM), DF was significantly lower than at baseline during stretch. After drug washout, a stretch-related increase in DF was again observed (pre 15 ± 4 vs. stretch 19 ± 3 Hz, p < 0.05). Myocardial stretch also produced a significant decrease in the VF spectral concentration (SpC), both at baseline (pre 31 ± 11 vs. stretch 22 ± 5, p < 0.05) and during 0.35 μM perfusion (pre 30 ± 9 vs. stretch 23 ± 6, p < 0.05), while at higher concentrations the variation did not reach statistical significance (0.7 μM pre 28 ± 7 vs. stretch 25 ± 5, ns; 1.4 μM pre 35 ± 11 vs. stretch 38 ± 12, ns). Spectral concentration during stretch was higher under the action of eleclazine 1.4 μM, and given their direct relationship with SpC, arrhythmia regularity and organization were also greater under the action of the drug.

### 3.3 Activation maps during VF

**FIGURE 5:**
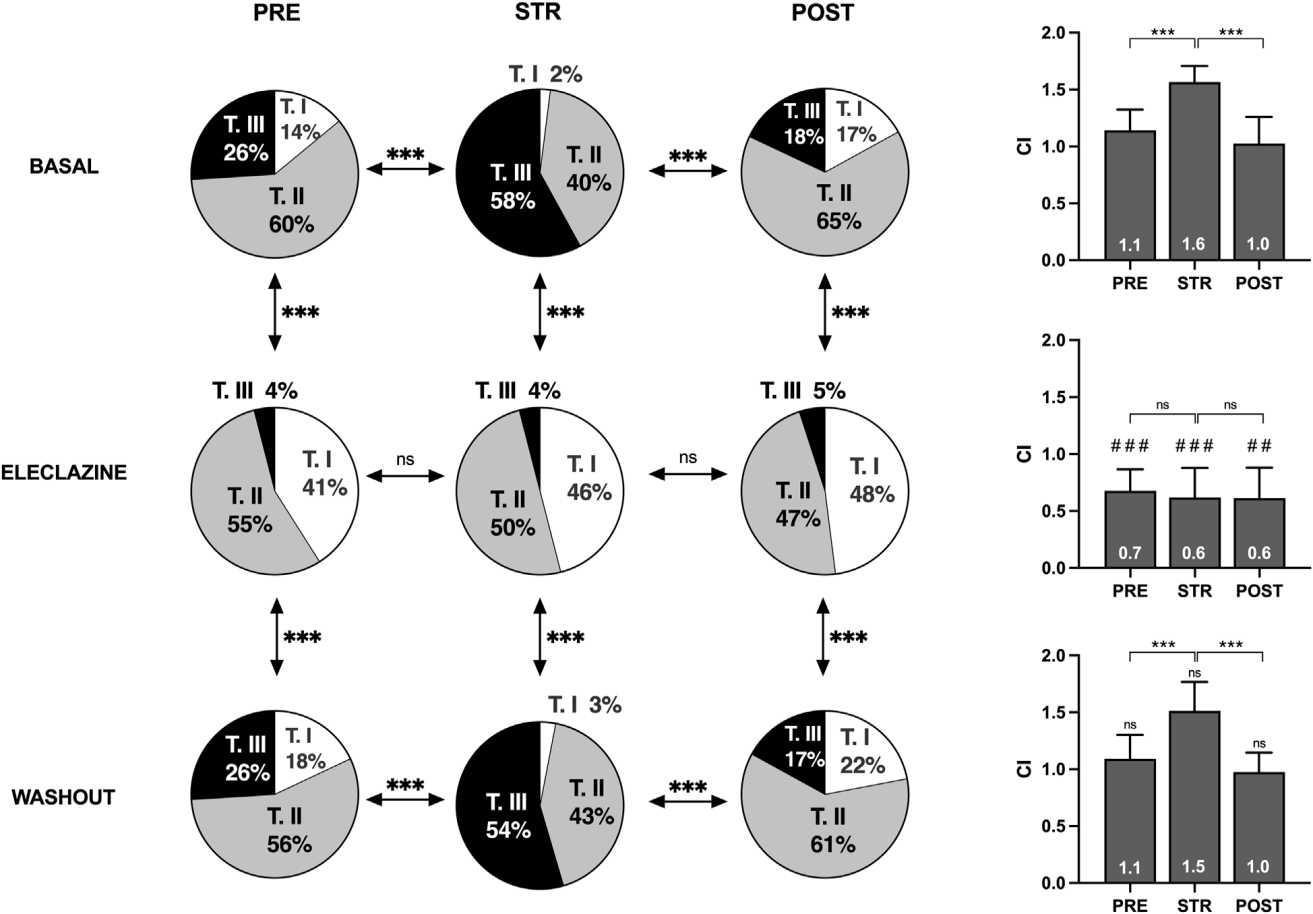
Percentages of activation maps and complexity index (CI) of VF obtained at baseline, during the perfusion of eleclazine 1.4 mM, and after drug washout before (PRE), during (STR) and after (POST) stretch (TI, TII and TIII = types of activation maps classified by complexity). Differences: ***p < 0.001. Differences vs. baseline: ##p < 0.01; ###p < 0.001.

**FIGURE 6:**
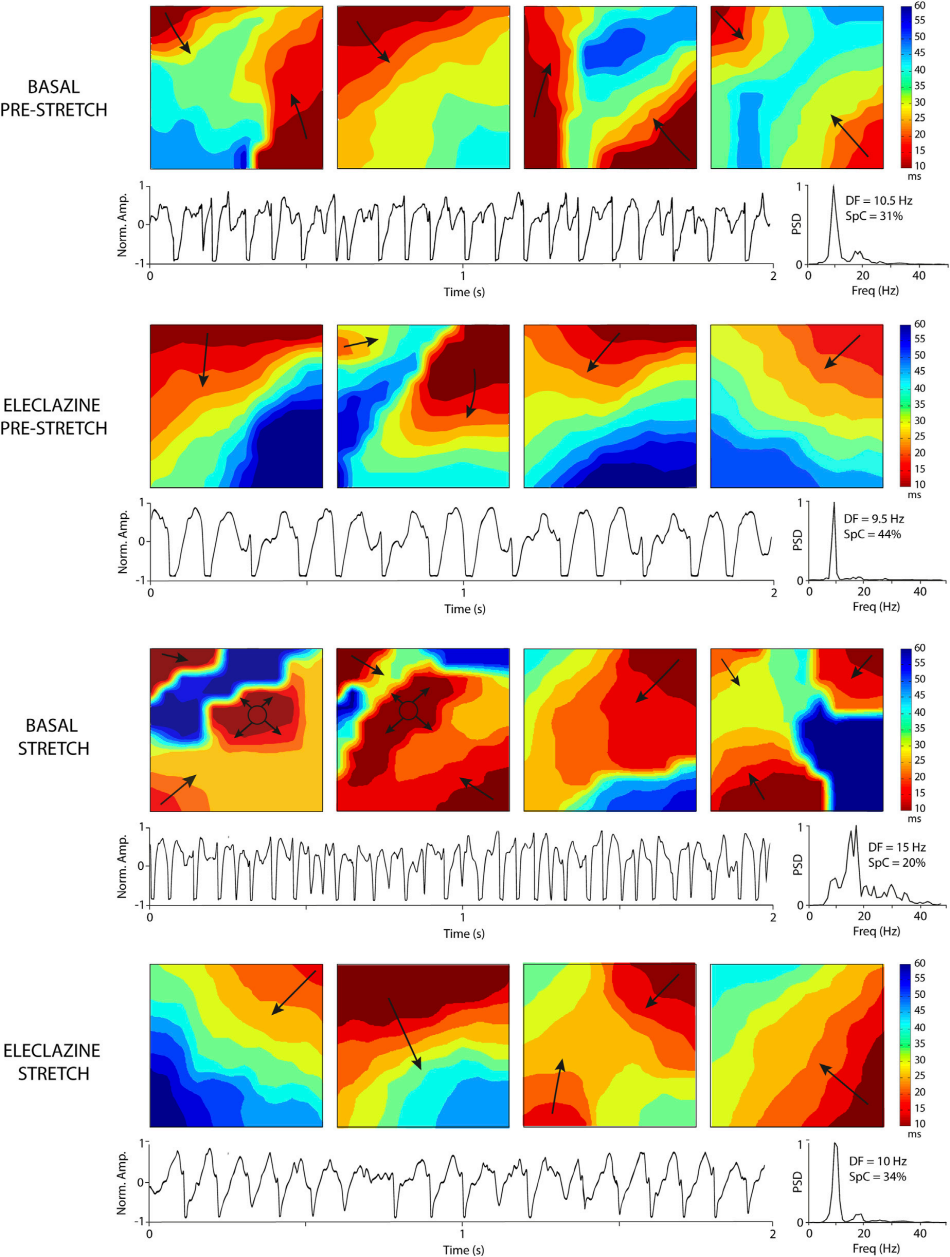
Activation maps and electrograms during VF obtained at baseline and under the perfusion of eleclazine 1.4 mM (before and during stretch). The increase in DF and complexity of the maps produced by stretch is not observed under the action of eleclazine. Abbreviations: DF, dominant frequency; Freq, frequency in hertz; Norm. Amp., normalized amplitude; PSD, Power spectral density; SpC = spectral concentration.

An analysis was made of the VF activation maps obtained at baseline and during perfusion at 1.4 μM - the concentration at which no significant stretch-induced variations in electrophysiological parameters are observed. The stretch-induced increase in complexity of the VF activation patterns observed at baseline was absent under the influence of the drug (n = 11) (Figures 5, 6). In parallel, therefore, the increase in type III maps and the decrease in type I maps observed at baseline during stretch was not found under the action of eleclazine, as reflected by the absence of significant variations in the VF complexity index (Such-Miquel et al., 2013): CI = (number of type I maps x 0.1 + number of type II maps × 1 + number of type III maps × 2)/(number of maps analyzed). Under the action of the drug, the activation maps were simpler than those obtained before, during or following the suppression of myocardial stretch. Complexity was similar to baseline after drug washout, and again the effects of stretch translated into an increase in arrhythmia complexity (baseline: CI pre 1.1 ± 0.2 vs. stretch 1.6 ± 0.1, p < 0.001; 1.4 μM: CI pre 0.7 ± 0.2 vs. stretch 0.6 ± 0.3, ns; and washout: CI pre 1.1 ± 0.2 vs. stretch 1.5 ± 0.2, p < 0.001).

### 3.4 Determinants of VF complexity

We further analyzed the association between arrhythmia complexity and the electrophysiological parameters obtained during VF. It was found that eleclazine 1.4 μM significantly lowered conduction velocity, while stretch produced no significant variations (Table 1). However, this parameter was not significantly modified under the action of eleclazine when it was determined during ventricular activation at a lower frequency (basic cycle of 250 ms). Correlations between the arrhythmia complexity index and the parameters P5 (r = −0.64), conduction velocity (r = 0.48) and wavelength during VF (r = −0.44) were significant (p < 0.001). The variable entering the equation in the multivariate analysis was P5. During the experimental protocol, VF stopped spontaneously under the action of eleclazine in 6 experiments. Cessation occurred during eleclazine 1.4 μM perfusion in 5 cases and at a 0.7 μM concentration in one case. At baseline, spontaneous cessation of arrhythmia was not observed in any of the cases studied during induced VF (n = 11) (p < 0.05).

**TABLE 1:** Conduction velocity determined at baseline, during the perfusion of eleclazine, and after drug washout.

		Baseline	Eleclazine 0.35 μM	Eleclazine 0.7 μM	Eleclazine 1.4 μM	Washout
CV-VF (cm/s)	PRE	49.0 ± 6.2	51.5 ± 2.7	49.6 ± 5.3	39.5 ± 7.4^###^	48.5 ± 3.6
	STR	48.6 ± 6.8	51.1 ± 2.0	50.6 ± 5.6	40.6 ± 3.9^##^	48.9 ± 5.5
	POST	49.6 ± 5.8	49.8 ± 6.3	49.3 ± 6.2	39.6 ± 5.3^###^	47.0 ± 3.4
CV-BASIC CYCLE (cm/s)	PRE	75.9 ± 7.1	76.2 ± 7.5	78.5 ± 6.7	76.9 ± 7.2	81.4 ± 7.5
	STR	77.8 ± 8.3	76.6 ± 8.2	79.4 ± 7.6	79.4 ± 7.7	79.9 ± 7.4
	POST	80.1 ± 7.8	79.3 ± 6.9	78.1 ± 8.2	77.9 ± 6.5	81.7 ± 7.8

Mean ± standard deviation of conduction velocity determined at baseline, during the perfusion of eleclazine (0.35, 0.7 and 1.4 μM), and after drug washout before (PRE), during (STR), and after (POST) stretch. Abbreviations: CV-VF, conduction velocity during VF; CV-BASIC CYCLE, conduction velocity during ventricular pacing at a basic cycle length of 250 m. Differences vs. baseline: ##p < 0.01; ###p < 0.001.

## 4 Discussion

**FIGURE 7:**
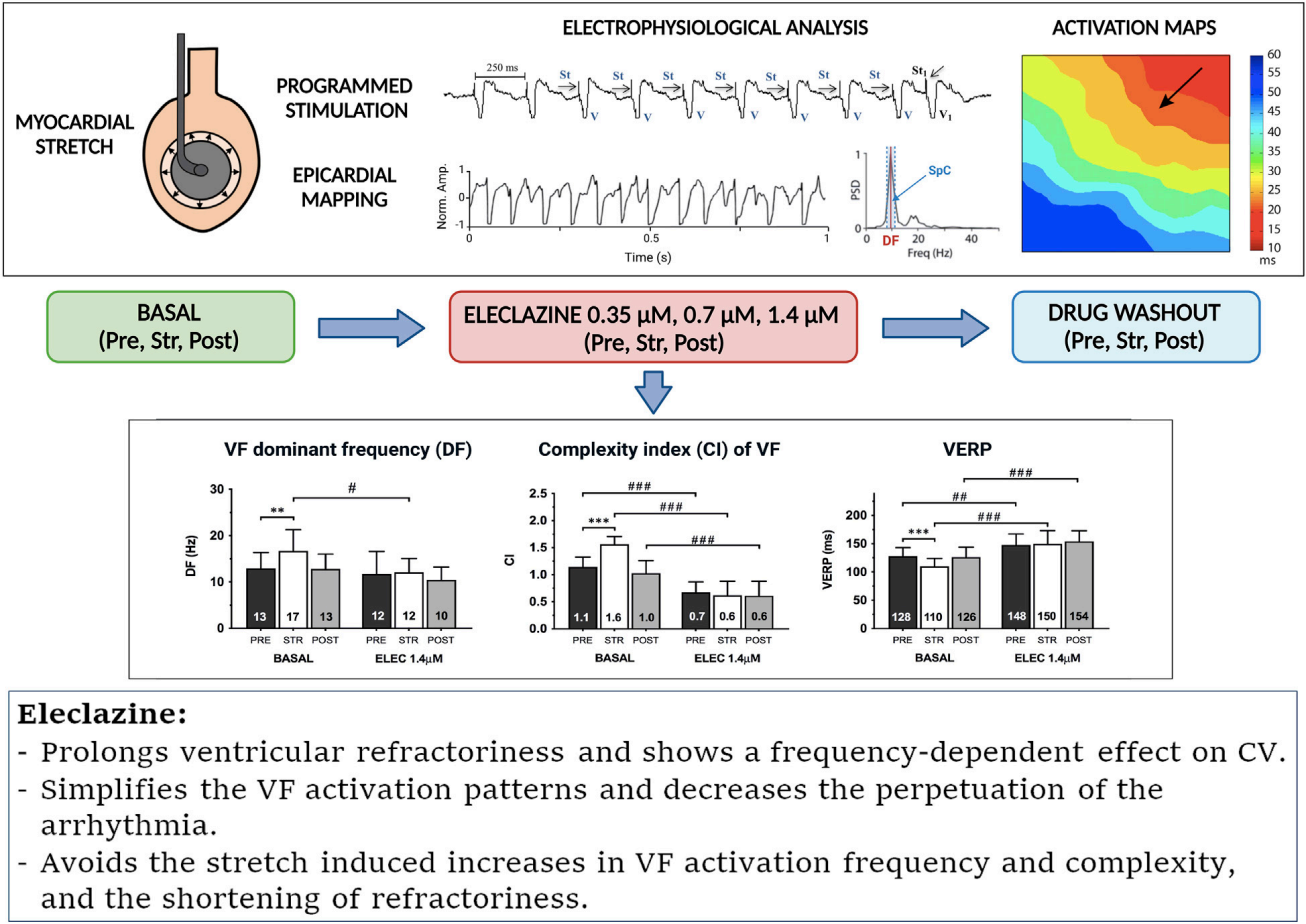
Summary of the methodology used and the effects of eleclazine on the electrophysiological modifications produced by myocardial stretch. Abbreviations: as in previous figures.

The main results of the present study indicate that eleclazine prolongs ventricular refractoriness, shows a frequency-dependent effect on conduction velocity, simplifies the activation patterns and decreases the perpetuation of the arrhythmia. The electrophysiological modifications induced by acute myocardial stretch are attenuated by eleclazine, which avoids the stretch-induced increases in VF activation frequency and complexity related to the stretch-induced shortening of refractoriness (Figure 7).

### 4.1 Effects of eleclazine on electrophysiological parameters and VF activation patterns

The effects of the INaL inhibitors ranolazine and GS 967 on basic electrophysiology have been studied in previous reports (Del-Canto et al., 2020; Chorro et al., 2015b; Milberg et al., 2013; Del Canto et al., 2018; Antzelevitch et al., 2004; Potet et al., 2016; Potet et al., 2020). Their actions have been related to the ability to inhibit INaL but also to the effects on the rapid delayed rectifier potassium current IKr and the consequent prolongation of refractoriness (Antzelevitch et al., 2004). On the other hand, the modifications induced in the rapid sodium current have also been implicated in the actions of both ranolazine and the more specific INaL inhibitors GS967 and eleclazine (Burashnikov and Antzelevitch, 2017; Caves et al., 2020; Potet et al., 2016; Potet et al., 2020).

In the present work, eleclazine has shown both a refractoriness-prolonging effect and a conduction velocity-reducing effect during VF. The concentrations used are in a range of the plasma concentrations achieved with therapeutically effective doses (Bacic et al., 2017). Interestingly, the effect on CV has not been observed during stimulation at a cycle length of 250 m and yet it has been significant during VF, when the activation frequency is faster. A use-dependent effect of INaL inhibitors GS-967 and eleclazine has been described in preparations of isolated rat cardiomyocytes (Caves et al., 2020), in heterologously expressed human Nav1.5 (Potet et al., 2016), and in human pluripotent stem-cell derived cardiomyocytes (Potet et al., 2020). Although eleclazine shows a preferential effect for INaL (El-Bizri et al., 2018), it also modifies the rapid component of the sodium current at higher concentrations (Caves et al., 2020; Potet et al., 2020; El-Bizri et al., 2018). In isolated rat myocytes (Caves et al., 2020) the inhibitory effects of eleclazine 10 μM on the rapid component of the sodium current showed a use-dependent effect, and this effect was also shown when using whole rat hearts. A use-dependent block of the rapid INa was also demonstrated in human pluripotent stem-cell derived cardiomyocytes, and the calculated IC50 for the use-dependent block was 0.6 μM at 10 Hz (Potet et al., 2020). The present study has shown a reduction in CV during VF recorded in whole rabbit heart preparations, and significant effects on the perpetuation of the arrhythmia. Both effects are compatible with a use-dependent effect on the rapid component of INa, an effect that at rapid activation frequencies occurs at drug concentrations closer to those at which INaL is inhibited (Burashnikov and Antzelevitch, 2017). The CV reduction in the context of VF has determined that the wavelength of the activation process during the arrhythmia has not been significantly modified during the 1.4 μM concentration despite the concomitant prolongation of ventricular refractoriness. However, the VF activation maps have been simpler and the VF complexity index has decreased, and the multivariate analysis has shown that the main determinant of this simplification of activation maps during the arrhythmia has been the ventricular refractoriness.

### 4.2 Eleclazine and mechanical cardiac stretch

Myocardial stretch is an arrhythmogenic factor (Quinn and Kohl, 2021; Sutherland, 2017; Johnson and Antoons, 2018; Gottlieb et al., 2023) that has been seen to modify the resting potential, duration of the action potential, myocardial refractoriness, and arrhythmia inducibility in several experimental models (Quinn and Kohl, 2021; Johnson and Antoons, 2018; Ravelli and Allessie, 1997; Chorro et al., 2005; Chorro et al., 2009; Reiter et al., 1988). The myocardial action potential characteristics vary according to transmembrane ionic current modifications (Kistamás et al., 2021; Grandi and Herren, 2014; Hegyi et al., 2018; Wagner et al., 2006), and stretch modifies the balance between inward and outward currents. Stretch increases Na^+^ and Ca^2+^ entry into the cardiac myocytes by opening stretch-sensitive channels (both K^+^ selective and non-cation selective), as well as by modifying the activity of Na^+^ channels and activating the Na^+^/Ca^2+^ exchanger in its reverse mode (Quinn and Kohl, 2021; Prosser et al., 2013; Neves et al., 2016). It modifies Nav1.5 channel function through mechanical action and by increasing reactive oxygen species (ROS) that either directly or with the intervention of protein kinase A and calcium calmodulin kinase II (CaMKII) delay their inactivation (Quinn and Kohl, 2021; Prosser et al., 2013; Ma et al., 2012). CaMKII is also influenced by the increase in Ca^2+^ and modifies the outflow currents of K^+^, ICaL, and INaL, contributing to the persistence of INaL along the action potential (Ma et al., 2012; Karagueuzian et al., 2017; Greer-Short et al., 2020; Wei et al., 2017; Shryock et al., 2013).

INaL activity is higher under conditions of oxidative stress or abnormalities in the management of intracellular Ca^2+^ (Hegyi et al., 2018; Sutanto et al., 2020; Horváth et al., 2023; Horváth et al., 2022; Kiss et al., 2021; Johnson, 2020; Takla et al., 2020; Belardinelli et al., 2015). In turn, the activity of the sarcoplasmic reticulum, its Ca^2+^ content, and the open probability of Ryanodine channels favoring the spontaneous release of Ca^2+^, are also dependent on sodium currents and calcium homeostasis (Kistamás et al., 2021; Takla et al., 2020). INaL inhibition (Ma et al., 2012; Karagueuzian et al., 2017) may contribute to a decrease in Na^+^ and Ca^2+^ entry, and in turn reduces some of their effects, such as diastolic depolarization, the dispersion of refractoriness, or increased ectopic activity (Kistamás et al., 2021; Karagueuzian et al., 2017; Horváth et al., 2022; Yamazaki et al., 2022). The regulation of the stretch-induced increase in Na + inflow and the activation of the reverse mode of the Na^+^/Ca^2+^ exchanger would contribute to attenuate the effects of increased intracellular Ca^2+^ on CaMKII which in turn phosphorylates the Na + channel and causes an increase in INaL. The reduction in Ca^2+^ concentration would also influence SERCA and the activation of the Ryanodine receptor, which increases the open probability of this receptor and the Ca^2+^ release from the sarcoplasmatic reticulum. Similar effects on the stretch-induced modifications of mechanoelectric feedback have been described on modifying Na^+^ and Ca^2+^ homeostasis through inhibition of the Na^+^/Ca^2+^ exchanger (Chorro et al., 2009) or the Na^+^/H^+^ exchanger (Chorro et al., 2015a).

On the other hand, eleclazine also has weak effects on other ion currents, and at a concentration of 14.2 μM it exerts a weak inhibitory effect upon IKr (Rajamani et al., 2016; Caves et al., 2020). As previously commented, eleclazine shows a preferential effect upon INaL (El-Bizri et al., 2018), but also modifies the rapid component of the sodium current especially at high activation frequencies, showing a use-dependent effect (Caves et al., 2020; Potet et al., 2020; El-Bizri et al., 2018). The effects on both components of the Na^+^ current and on IKr probably play a role in the decreased conduction velocity and increased refractoriness observed under the action of this drug and in the clear attenuation of the stretch-induced effects observed in this study. In this regard, Rajamani et al. (2016) analyzed the magnitude of inhibition of the rapid component of INa by eleclazine 10 μM at two stimulation frequencies in rabbit ventricular myocytes. The inhibitory effect was around 10% at a stimulation frequency of 0.1 Hz and around 20% at a frequency of 3 Hz. They also observed that eleclazine 2 μM, a concentration that is twice the clinical therapeutic concentration, caused less than 10% decrease in the maximum rate of depolarization of ventricular action potential at stimulating frequencies of 60, 180 and 210 bpm. El-Bizri et al. (2018) in human cardiac voltage-gated sodium channels reported an IC50 value of 0.62 ± 0.12 μM to inhibit INaL, with a high selectivity with respect to the effects over the rapid component of INa. Caves et al. (2020) reported an IC50 of 179.9 nM to inhibit the activated INaL in rat ventricular myocytes, and they also observed that eleclazine 10 μM inhibited rapid INa in a use-dependent manner. Potet et al. (2020) reported a reduction of the rapid component of INa in a frequency-dependent manner, and the IC50 determined in human induced pluripotent stem-cell derived cardiomyocytes was 0.6 μM at 10 Hz. They related the use-dependent effect to the high affinity binding to the receptor and a slowed recovery from inactivation of the rapid sodium current. The sodium-channel blocking activity may delay the recovery of excitability prolonging refractoriness. Thus, the inhibition of the rapid component of INa, the reduction of action potential phase 0 slope, the presence of post-repolarization refractoriness and the consequent flattening of the steep initial portion of the restitution curve of refractoriness are factors that may have contributed to simplifying the VF activation patterns.

### 4.3 Clinical implications

Myocardial fiber stretch and its proarrhythmic action is present in various situations, including regional contraction anomalies secondary to conduction disorders, ischemia or arrhythmias, and mitral prolapse, as well as acute pressure or volume overloads in various clinical contexts (Quinn and Kohl, 2021; Sutherland, 2017; Johnson and Antoons, 2018; Gottlieb et al., 2023; Barber et al., 2020). A reduction in the proarrhythmic effects of stretch via blockade of Na^+^/Ca^2+^ and Na^+^/H^+^ exchangers or beta-adrenergic receptors has been described in various experimental studies (Chorro et al., 2009; Chorro et al., 2015a). Beneficial effects have also been demonstrated in INaL inhibition with ranolazine in experimental models and in the clinical setting, where atrial and ventricular arrhythmia-reducing effects have been observed (Del-Canto et al., 2020; Chorro et al., 2015b; Younis et al., 2022). However, a reduction in arrhythmic complications has not been demonstrated in clinical studies with eleclazine in patients with Na^+^ channel alterations such as long QT syndrome type 3 or hypertrophic cardiomyopathy (Shen et al., 2022; Olivotto et al., 2016). The results of the present study show a decrease in the effects of stretch in the experimental model used and suggest a protective role in these settings. Nevertheless, interspecies differences must be taken into account when extrapolating the results obtained to humans. The electrophysiological characteristics of the myocytes of rabbits are closer to those of humans than those of mice and rats. Repolarization involves K+ ionic currents in which expression is similar in the ventricular myocardium of rabbits and humans. On the other hand, the contribution of ICaL and the Na^+^/Ca^2+^ exchanger to calcium homeostasis is higher in humans and rabbits than in mice and rats, and also there are differences in the activity of the sarcoplasmic reticulum and the sarcoplasmic reticulum calcium ATPase (SERCA). Differences in calcium handling, action potential restitution and heart size determine a lower susceptibility to electrical alternans phenomena in the experimental models in which mice and rats are used. For these reasons, when considering the clinical implications, it must be kept in mind that experimental models differ from clinical settings. The experimental model used in the present work allows the stretching protocol to be carried out in a reproducible manner and thus study the influence of specific variables. The main objective has been to characterize the modifications of the electrophysiological effects produced by the acute stretching of the myocardium under the action of the drug. This objective is in line with previous studies in which pathophysiological information related to the mechanoelectric feedback has been provided (Chorro et al., 2005; Chorro et al., 2009; Chorro et al., 2015a; Del-Canto et al., 2020; Chorro et al., 2015b; Del Canto et al., 2018).

### 4.4 Limitations

Interspecies differences must be taken into account when extrapolating the results obtained to the clinical setting. Moreover, the methodology applied in this study involves acute myocardial stretch, and the effects of stretch can give rise to different manifestations in chronic preparations. The effects of stretch in chronic preparations can lead to different manifestations, given the influence of other factors, among them the associated neurohumoral reflexes, the structural changes elicited by the chronic stretch or the electrophysiological remodeling. Neurohumoral reflexes can modulate the direct effects of stretch on the myocardium. On the other hand, the administration of the drug in chronic settings, may add other effects not elicited during the acute administration. In general terms, the acute models facilitate the control of some variables that influence the results, while chronic studies allow us to get closer to the clinical settings.

## 5 Conclusion

The late sodium current inhibitor eleclazine (GS6615) decreased the proarrhythmic electrophysiological changes induced by myocardial stretch in the experimental model used, and significantly altered activation patterns during VF.

## Data Availability

The raw data supporting the conclusions of this article will be made available by the authors, without undue reservation.

## Abbreviations

CaMKII, calcium calmodulin kinase II; CI, ventricular fibrillation complexity index; CV, conduction velocity DF, ventricular fibrillation dominant frequency; IKr, rapidly activating delayed rectifier potassium current; INa, sodium current; INaL, late sodium current; P5, 5^th^ percentile of VV intervals during ventricular fibrillation; ROS, reactive oxygen species; SpC, spectral concentration; VF, ventricular fibrillation.
